# Drug library screening identifies histone deacetylase inhibition as a novel therapeutic strategy for choriocarcinoma

**DOI:** 10.1002/cam4.5243

**Published:** 2022-09-15

**Authors:** Eri Watanabe, Akira Yokoi, Kosuke Yoshida, Mai Sugiyama, Masami Kitagawa, Kimihiro Nishino, Eiko Yamamoto, Kaoru Niimi, Yusuke Yamamoto, Hiroaki Kajiyama

**Affiliations:** ^1^ Department of Obstetrics and Gynecology Nagoya University Graduate School of Medicine Nagoya Japan; ^2^ Institute for Advanced Research Nagoya University Nagoya Japan; ^3^ Bell Research Center, Department of Obstetrics and Gynecology Collaborative Research Nagoya University Graduate School of Medicine Nagoya Japan; ^4^ Department of Healthcare Administration Nagoya University Graduate School of Medicine Nagoya Japan; ^5^ Laboratory of Integrative Oncology National Cancer Center Research Institute Tokyo Japan

**Keywords:** choriocarcinoma, drug repositioning, ferroptosis, histone deacetylase inhibitors, vorinostat

## Abstract

**Background:**

Choriocarcinoma is a rare and aggressive gynecological malignancy. The standard treatment is systemic chemotherapy as choriocarcinoma exhibits high chemosensitivity. However, refractory choriocarcinoma exhibits chemoresistance; thus, the prognosis remains very poor. This study aimed to identify novel therapeutic agents for choriocarcinoma by utilizing a drug repositioning strategy.

**Methods:**

Three choriocarcinoma cell lines (JAR, JEG‐3, and BeWo) and a human extravillous trophoblast cell line (HTR‐8/SVneo) were used for the analyses. The growth inhibitory effects of 1,271 FDA‐approved compounds were evaluated in vitro screening assays and selected drugs were tested in tumor‐bearing mice. Functional analyses of drug effects were performed based on RNA sequencing.

**Results:**

Muti‐step screening identified vorinostat, camptothecin (S, +), topotecan, proscillaridin A, and digoxin as exhibiting an anti‐cancer effect in choriocarcinoma cells. Vorinostat, a histone deacetylase inhibitor, was selected as a promising candidate for validation and the IC50 values for choriocarcinoma cells were approximately 1 μM. RNA sequencing and subsequent pathway analysis revealed that the ferroptosis pathway was likely implicated, and key ferroptosis‐related genes (i.e., *GPX4*, *NRF2*, and *SLC3A2*) were downregulated following vorinostat treatment. Furthermore, vorinostat repressed tumor growth and downregulated the expression of GPX4 and NRF2 in JAR cell‐bearing mice model.

**Conclusion:**

Vorinostat, a clinically approved drug for the treatment of advanced primary cutaneous T‐cell lymphoma, showed a remarkable anticancer effect both in vitro and in vivo by regulating the expression of ferroptosis‐related genes. Therefore, vorinostat may be an effective therapeutic candidate for patients with choriocarcinoma.

## Graphical abstract

We evaluated the growth inhibitory effect of 1271 FDA‐approved compounds by MTS assay and identified vorinostat, a histone deacetylase inhibitor, as one of the therapeutic candidates. Vorinostat showed a remarkable anti‐cancer effect both in vitro and in vivo by inducing ferroptosis.

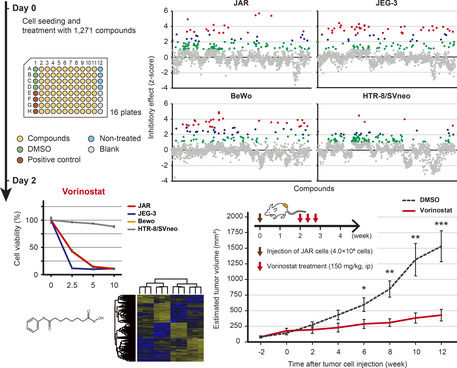

## 1 INTRODUCTION

Choriocarcinoma is the most aggressive gestational trophoblastic disease (GTD), which is characterized by aberrant proliferation of atypical placental trophoblasts. The incidence of choriocarcinoma ranges from 1 to 9.2 in 40,000 pregnancies.^1^ The specific marker for choriocarcinoma is human chorionic gonadotropin (hCG), which is used to evaluate response to treatment and to diagnose recurrence. Choriocarcinoma can develop after all forms of pregnancy, including complete hydatidiform mole (CHM), partial hydatidiform mole (PHM), and abortion.^2^ CHM accounts for 50% of all choriocarcinoma cases.^3^ Typically, CHM has two paternal genomes, whereas PHM is triploid of both maternal and paternal genetic origin.^4^ The underlying genetic characteristics and molecular pathology is complicated and remains unclear. Chemotherapy, including methotrexate, etoposide, and actinomycin D, is the primary treatment option for choriocarcinoma.^5^ In general, the chemosensitivity of choriocarcinoma is high; therefore, approximately 90% of the patients achieve a response following initial treatment.^6^, ^7^ However, the prognosis is poor for the remaining 10% who develop metastasis and acquired resistance. Therefore, it is important to identify novel therapeutic agents for these patients.^6^

Recently, high‐throughput methodologies, such as next‐generation sequencing, have been developed, and target‐based screening is a strategy to identify novel therapeutic agents.^8^ However, target‐based screening has been less successful than expected.^9^ Thus, an alternative method is a phenotype‐based approach, including research using small molecule libraries.^10^, ^11^ The Prestwick Chemical Library® consists of 1271 compounds with well‐annotated pharmacology. Moreover, they are clinically approved by the Food and Drug Administration (FDA), European Medicines Agency (EMA), and other agencies. Therefore, the toxicity is considered to be well‐tolerated in humans. This is a significant advantage in the development of drugs for rare cancers, including choriocarcinoma.

In this study, we identified novel therapeutic agents based on a drug repositioning strategy using three choriocarcinoma cell lines. Vorinostat, a histone deacetylase (HDAC) inhibitor, decreased cell proliferation both in vitro and in vivo. Then, RNA sequencing suggested that vorinostat may induce ferroptosis in choriocarcinoma cells.

## 2 MATERIALS AND METHODS

### 2.1 Cell culture

The human choriocarcinoma cell lines, JAR, JEG‐3, and BeWo, were purchased from the American Type Culture Collection. The human extravillous trophoblast cell line, HTR‐8/SVneo, was provided by Dr. Charles H. Graham (Queen's University). Cells were maintained in RPMI 1640 (Nacalai tesque) supplemented with 10% fetal bovine serum (FBS) and antibiotics.

### 2.2 Measurement of hCG in the culture supernatant

To measure hCG protein levels in a conditioned medium, cells were seeded into 60 mm plates and cultured for 48 h. The culture supernatant was collected and hCG levels were measured by SRL, Inc.

### 2.3 Immunofluorescence

The cells were fixed with 4% paraformaldehyde. Then, cells were blocked with 7% FBS/PBS at room temperature for 1 h and permeabilized with 0.5% Triton X‐100 in PBS at room temperature for 10 min. Next, the samples were incubated with anti‐hCG primary antibody (HCG‐55, Novus Biologicals LLC) in 7% FBS/PBS at 4°C overnight. The samples were incubated with Alexa Fluor 488‐conjugated secondary antibody (Thermo Fisher Scientific) and 4′,6‐diamidino‐2‐phenylindole for 1 h. Finally, the samples were analyzed with a Keyence BZ‐X800 (KEYENCE).

### 2.4 Cell proliferation assay

The Prestwick Chemical Library (Prestwick Chemical), which contains 1271 small molecules, was used. About 95% of the compounds are clinically approved drugs. Vorinostat was obtained from ChemShene, LLC (Monmouth Junction). Drugs were dissolved in DMSO for each analysis.

Cells were seeded into 96‐well plates at 8000 cells/well (JAR) or 10,000 cells/well (JEG‐3, BeWo, HTR8/SVneo). Twenty‐four hour after seeding, drugs were added to each well at a concentration of 10 μM. After 48 h, cell viability was measured using the CellTiter 96 Aqueous One Solution cell proliferation assay kit (Promega). The optical density was determined using a microplate reader (Agilent Technologies). Cells exposed to vorinostat (1 μM) were evaluated in real‐time for cell confluence using IncuCyte ZOOM equipment (Essen BioScience). The percentage of confluence was analyzed by IncuCyte ZOOM software.

### 2.5 RNA sequencing and pathway analysis

Cells were treated with either 1 μM vorinostat or DMSO for 24 h. Total RNA was extracted from JAR and JEG‐3 cells using the RNeasy Plus Mini Kit (QIAGEN). Total RNA concentration was measured using a NanoDrop ND‐1000 spectrophotometer (Thermo Fisher Scientific). Rarevariant, Inc. conducted the RNA sequencing. Kallisto software was used to quantify the expression of each gene. Subsequently, the tximport package (ver. 1.18.0) of R software (ver. 4.0.3) and RStudio (RStudio) were used, and scaledTPM counts were calculated. Excluding genes with low read coverage, 1159 differentially expressed genes (DEGs, |log2FC| > 1) were used for heatmap and principal component analysis (PCA). The heatmap.2 function of the gplots package (ver. 3.1.0) and the prcomp and plot3d functions of the rgl package (ver. 0.100.54) were used. The Wald test in DESeq2 (ver. 1.30.0) was used to calculate adjusted *p*‐values for each gene, and the volcano plot was visualized. The Ingenuity Pathway Analysis (IPA, QIAGEN) program was performed using significant DEGs from a vorinostat‐treated JAR or JEG3 cells. Commonly dysregulated pathways following vorinostat treatment were identified using a Venn diagram.

### 2.6 qRT‐PCR

Total RNA was isolated as described above and cDNA was synthesized using a cDNA Reverse Transcription Kit (Toyobo). TB Green Premix Ex Taq (Takara Bio) was used. The primer sequences are shown in Table S1, and Mx3000P (Agilent Technologies) was used. The following amplification program was used: denaturation at 95°C for 10 min, followed by 40 amplification cycles of 95°C for 15 s and 60°C for 1 min. GAPDH was used as a loading control.

### 2.7 Western blot analysis

Total protein was prepared using lysis buffer (0.1% triton X, TBS, phosphoblocker). After the separation of the cell lysate proteins by SDS‐PAGE electrophoresis, the cells were transferred to nitrocellulose membranes and blocked with 5% non‐fat milk. The membranes were then incubated overnight at 4°C with the following primary antibodies: Anti‐GPX4 antibody AB125066 (Abcam), anti‐NRF2 antibody #12721 (Cell Signaling Technology), and anti‐β‐actin (Sigma–Aldrich, St Louis). After incubation for 1 h at room temperature with peroxidase‐conjugated secondary antibody (Jackson Immuno Research Laboratories), the protein bands were examined using an ECL Western Blotting Detection Reagent and ImageQuant LAS 4000 mini software (GE Healthcare).

### 2.8 Immunohistochemistry

Sections of 4‐μm thickness were prepared using a microtome. The sections were deparaffinized, rehydrated, and subjected to antigen removal in 10 mM citrate buffer (pH 6.0; GPX4) or Tris‐EDTA buffer (pH 8.0; NRF2) at 95°C for 15 min. Endogenous peroxidase activity was blocked with 3% H_2_O_2_ in methanol for 5 min at room temperature. The sections were blocked with 10% goat serum and incubated with primary antibodies at 4°C overnight. Anti‐GPX4 antibody AB125066 (Abcam), anti‐NRF2 antibody #12721 (Cell Signaling Technology), anti‐4HNE antibody bs6313R (Bioss), and anti‐Ki67 ab16667 (Abcam) were diluted at 1:250, 1:100, and 1:600, respectively with 1% BSA/PBS. Then, the sections were incubated for 5 min with secondary antibody and peroxidase‐labeled streptavidin using SAB‐PO kits (Nichirei Biosciences). The sections were developed using DAB (Nichirei Biosciences). Finally, the sections were incubated with hematoxylin, dehydrated, and mounted. TUNEL‐HRP‐DAB staining (ab206386) was performed according to the manufacturer's instructions. Images were captured using a whole slide scanner (OliVIA VS120: Olympus Corp.) and quantified using TissueMorph software (VisioPharm).

### 2.9 Animal studies

All animal procedures were reviewed and approved by the Nagoya University Institutional Animal Experimentation Committee (Approval No. 20392). Five‐week‐old female BALB/Slc‐nu/nu mice were purchased from Japan SLC, Inc. JAR cells (4.0 × 10^6^ cells/PBS) were injected subcutaneously on both flanks. When tumors became palpable, the mice were divided into two groups and drug treatment was initiated. The mice were treated with either 150 mg/kg of vorinostat or vehicle (0.1% DMSO) three times every other day. The tumor volume was measured every 2 days and calculated using the modified ellipsoid formula (Length × Width^2^ × 0.5). The weight of the mice was measured once a week.

### 2.10 Statistical analysis

Statical analysis was performed with GraphPad Prism ver. 5.00 software for Windows (GraphPad Software). Student's *t*‐test was used to determine the significance of differences between the means of two sets of data. Differences with *p* < 0.05 were considered statistically significant.

## 3 RESULTS

### 3.1 Primary screening of a chemical library using choriocarcinoma cells

**FIGURE 1:**
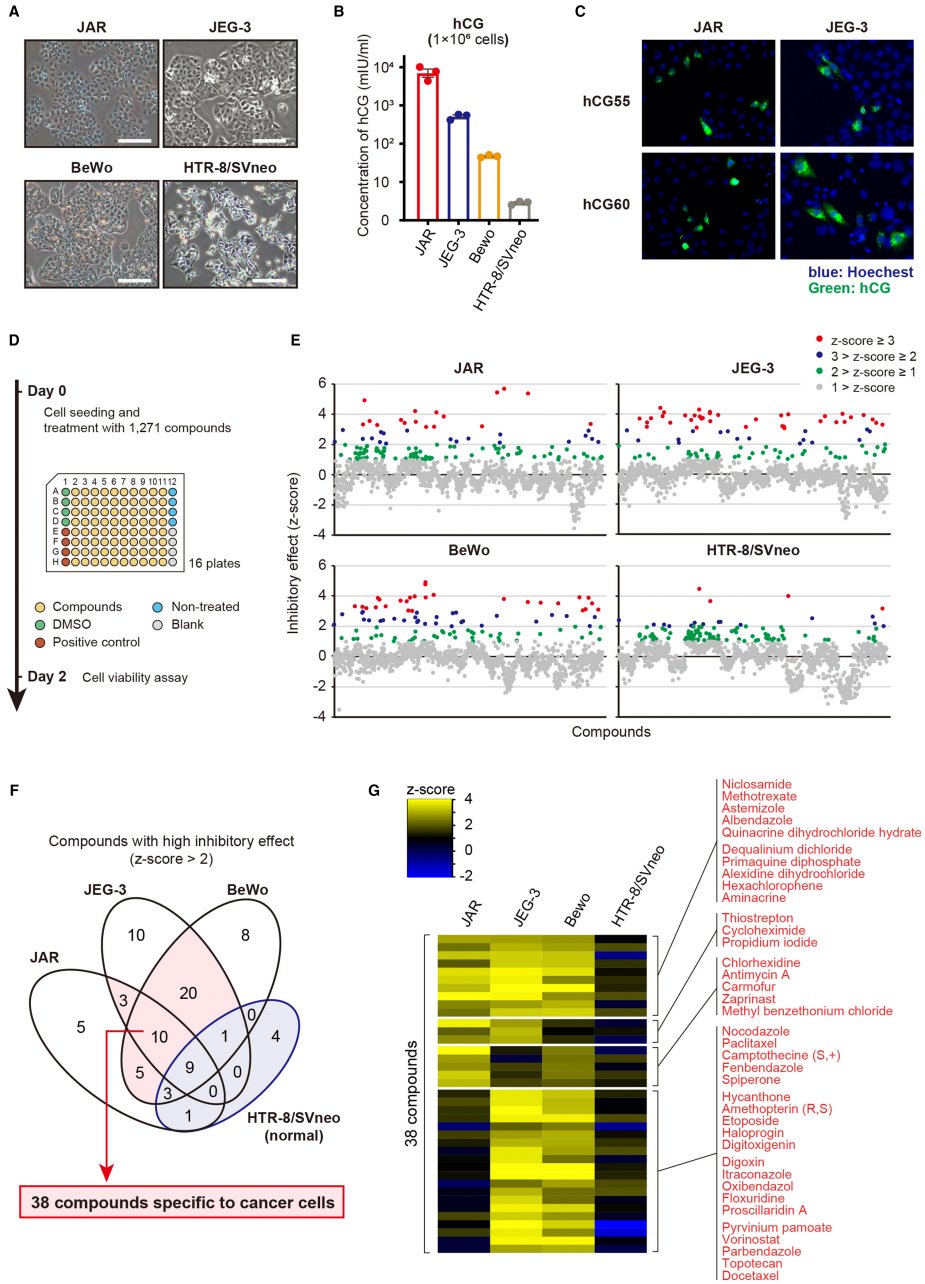
Primary screening to evaluate the relative inhibitory effects of 1271 compounds. (A) Representative images of choriocarcinoma (JAR, JEG‐3, and BeWo) and extravillous trophoblast (HTR‐8/SVneo) cell lines. Scale bar, 100 μm. (B) The levels of hCG in the culture medium of the cell lines. (C) Representative images of immunofluorescence staining of hCG in JAR and JEG‐3 cell lines. (D) Schematic overview of the screening protocol. (E) The *z*‐scores of the inhibitory effects of 1271 compounds in each cell line. (F) Venn diagram showing compounds with high inhibitory effects (*z*‐score > 2) in common. (G) Heatmap showing the compound with an inhibitory effect (*z*‐score > 1) in at least one cell line. The names of 38 compounds with high inhibitory effects are shown.

We used three choriocarcinoma cell lines (JAR, JEG‐3, BeWo) and one trophoblast cell line (HTR‐8/SVneo). Their representative images are shown in Figure 1A. Choriocarcinoma cell lines secreted more hCG into the culture medium compared with HTR‐8/SVneo cells, and immunofluorescence confirmed that hCG was highly expressed in JAR and JEG‐3 cells (Figure 1B,C). These results were consistent with the properties of choriocarcinoma cells.

First, we evaluated the relative growth inhibitory effect of 1271 compounds in each cell line using the screening procedure summarized in Figure 1D. Briefly, the cells were treated with 10 μM of each compound for 48 h. Growth inhibition was determined using an MTS assay. For each cell line, most of the compounds had no effect on cell viability, but several compounds exhibited relatively high growth inhibitory effects (Figure 1E). We evaluated the drugs with the high anticancer effects (*z*‐score > 2) on at least two cancer cell lines with a limited effect on the HTR‐8/SVneo cell line (*z*‐score ≤ 2). Using a Venn diagram, 38 compounds were selected (Figure 1F). A heatmap shows the names of the 38 compounds and their relative inhibitory effects on the cell lines (Figure 1G).

### 3.2 Identification of therapeutic candidates

**FIGURE 2:**
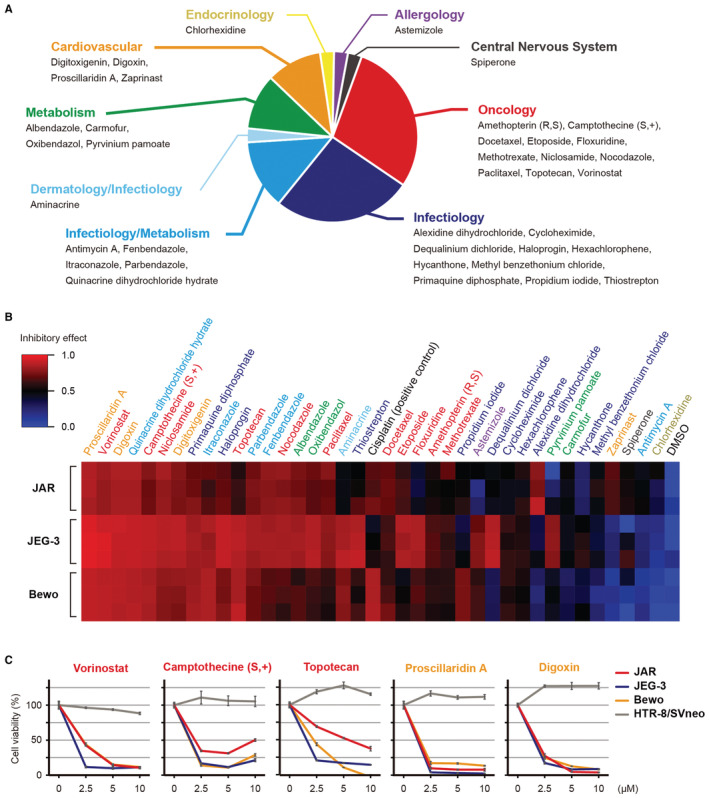
Secondary screening to identify therapeutic candidates. (A) Therapeutic category of the 38 compounds identified in the primary screen. (B) Heatmap showing the inhibitory effects of the 38 compounds in choriocarcinoma cell lines. Cells were treated with each compound in triplicate. Cisplatin and DMSO were used for positive and negative controls, respectively. (C) The efficacy of vorinostat, camptothecine (S, +), topotecan, proscillaridin, and digoxin in choriocarcinoma and extravillous trophoblast (HTR‐8/SVneo) cell lines. Cells were treated with each compound in triplicate and the data are presented as the mean ± standard error.

**FIGURE 3:**
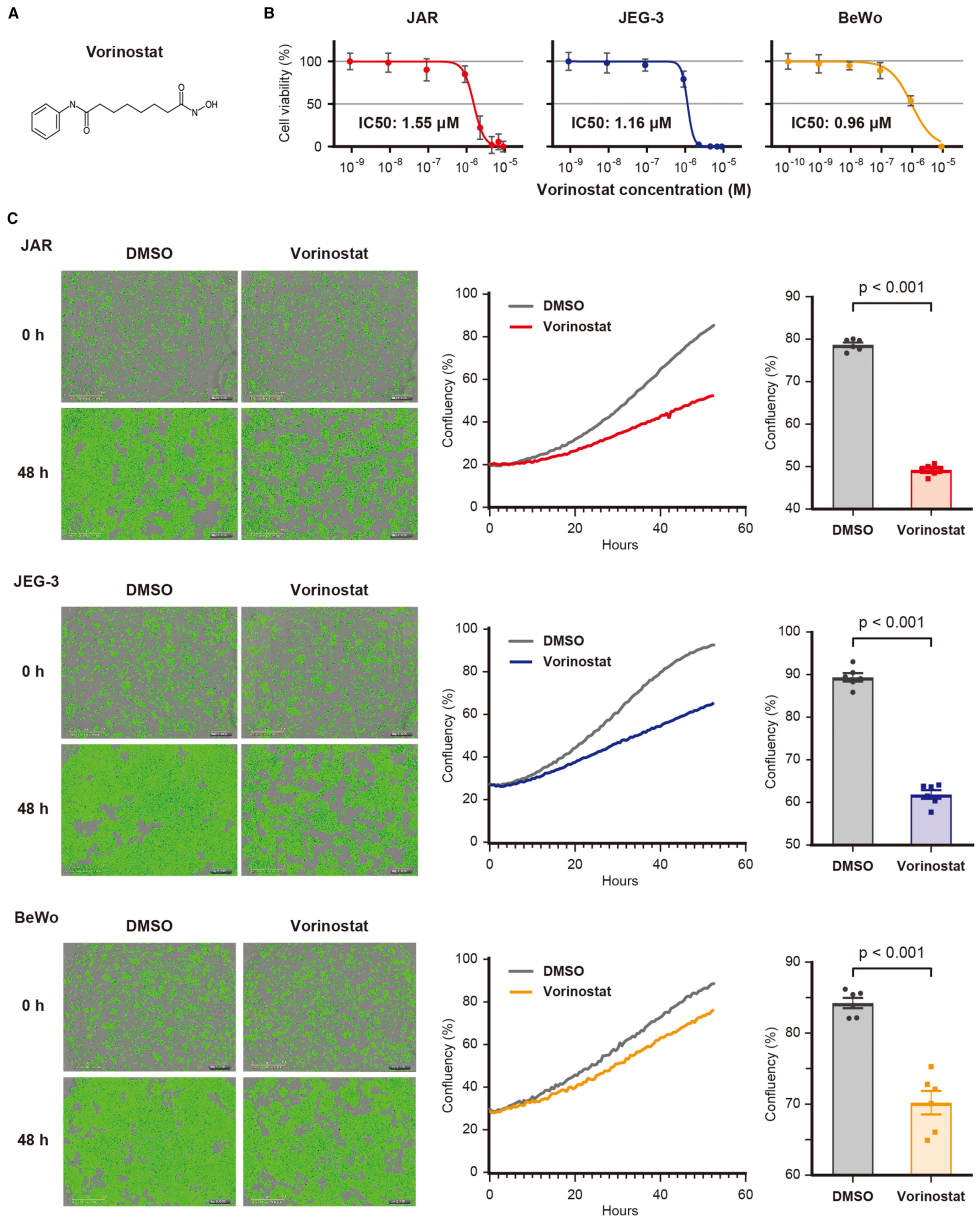
The effect of vorinostat on choriocarcinoma cell proliferation. (A) Structure of vorinostat. (B) The IC50s of vorinostat in three choriocarcinoma cells. (C) The time‐lapse imaging of vorinostat‐treated cells. Representative images of choriocarcinoma cells at 0 and 48 h (left), confluency of cells post‐treatment at 0–48 h (middle), and confluency of cells at 48 h (right) are shown. Cells were treated with either 1 μM vorinostat or DMSO, and confluency was compared by a Student's *t*‐test. The data consists of 6 technical replicates and the values are shown as the mean ± standard error of the mean.

Next, we classified the 38 drugs according to their expected pharmacological effects. Most of the drugs were associated with oncology or infectiology (Figure 2A). For the second screening, cells were treated with 10 μM of the 38 compounds for 48 h and the growth inhibition rate was evaluated. The results indicated that 19 compounds exhibited superior growth inhibition compared with cisplatin (a positive control). In particular, several oncological and cardiovascular drugs, including vorinostat, camptothecin (S, +), topotecan, proscillaridin A, and digoxin, exhibited significant effects in the choriocarcinoma cell lines (Figure 2B). We then examined the concentration‐dependent antitumor effects of these drugs. Consistent with the results of primary screening, these five drugs were effective even at low micromolar concentrations without affecting HTR‐8/SVneo viability (Figure 2C). Therefore, we considered these drugs as therapeutic candidates. However, camptothecin (S, +) and topotecan were excluded because the other topoisomerase inhibitor was already in clinical use for choriocarcinoma. Moreover, proscillaridin A and digoxin were excluded due to potential cardiac adverse events. Therefore, we focused on vorinostat, an HDAC inhibitor, for subsequent experiments (Figure 3A).

### 3.3 The effect of vorinostat on choriocarcinoma cells

The IC50 values of vorinostat in JAR, JEG‐3, and BeWo cells were 1.55, 1.16, and 0.96 μM, respectively (Figure 3B). The effect of vorinostat was validated by time‐lapse imaging (Figure 3C), showing that vorinostat treatment significantly inhibited cell proliferation compared with DMSO treatment (*p* < 0.05).

**FIGURE 4:**
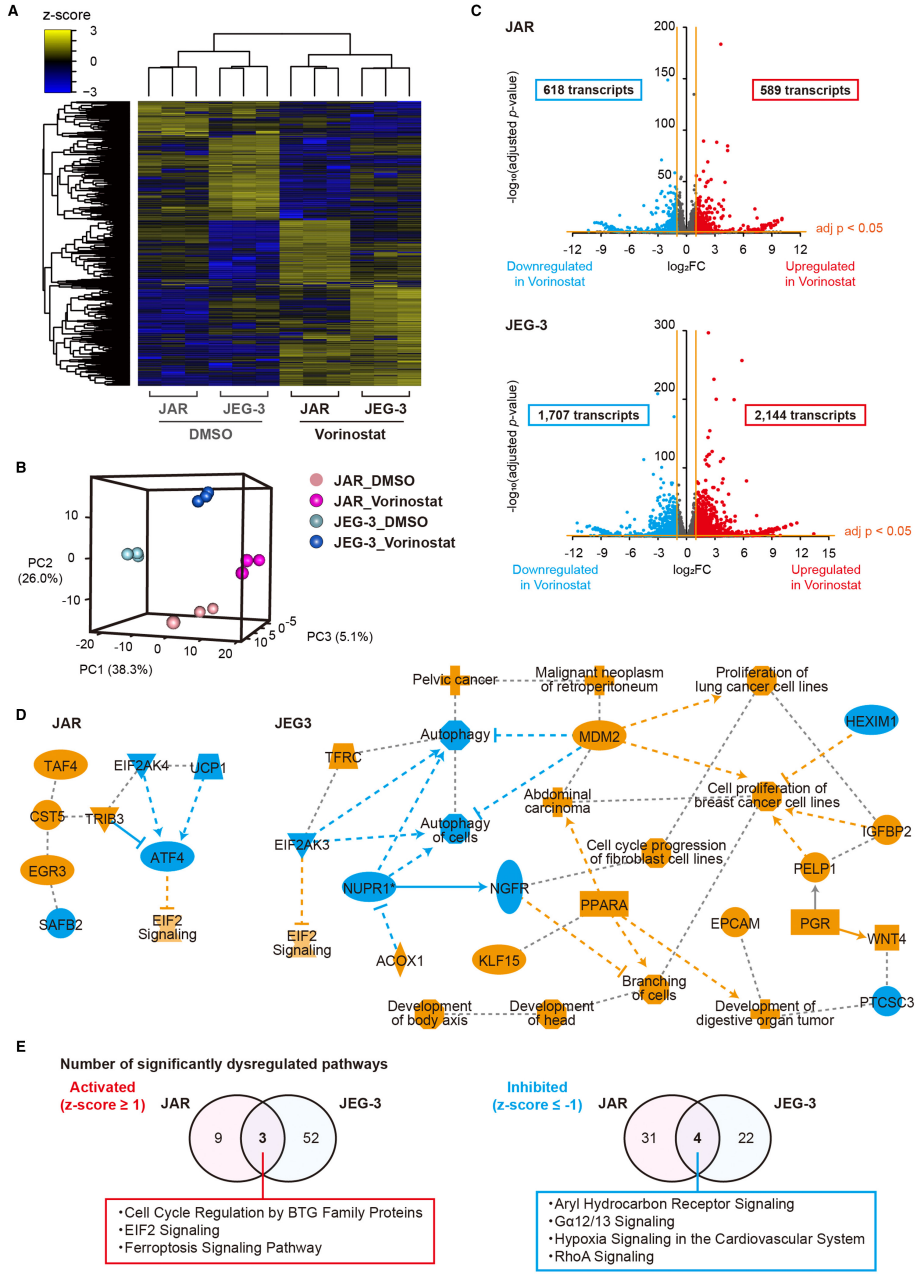
RNA sequencing to identify the mechanism of vorinostat‐induced cell death. (A) Heatmap analysis showing differentially expressed genes (GEGs, |log2FC| > 1) following vorinostat treatment. Choriocarcinoma cells were treated with either 1 μM vorinostat or DMSO for 24 h. (B) Principal component analysis of vorinostat‐ or DMSO‐treated choriocarcinoma cells. (C) Volcano plots of vorinostat‐ or DMSO‐treated choriocarcinoma cells. (D) Graphical summary based on Ingenuity Pathway Analysis (IPA). Orange and blue nodes showed activated and inhibited factors, respectively. (E) Venn diagrams representing the commonly activated or inhibited pathways following vorinostat treatment based on IPA analysis.

To investigate the potential mechanisms of vorinostat activity, we performed RNA sequencing using JAR and JEG‐3 cells. The heatmap and PCA analysis revealed that vorinostat‐treated cells exhibit different RNA profiles compared with DMSO‐treated cells (Figure 4A,B). A volcano plot showed that there were 1207 and 3851 significantly DEGs by vorinostat in JAR and JEG‐3 cells (Figure 4C and Tables S2 and S3). IPA analysis of the DEGs was performed for each cell line and a graphical summary is shown in Figure 4D. Moreover, the pathway analysis and Venn diagram revealed that three and four pathways were significantly activated and inhibited by vorinostat treatment in both cell lines, respectively (Figure 4E and Tables [Link], [Link], [Link], [Link]). Of these pathways, we focused on the ferroptosis signaling pathway (JAR and JEG‐3, *p* = 4.17e‐5 and 6.76e‐4, respectively). This is because choriocarcinoma clinically exhibited intratumoral hemorrhage, suggesting an iron‐abundant cancer microenvironment.

### 3.4 Activation of ferroptosis by vorinostat

**FIGURE 5:**
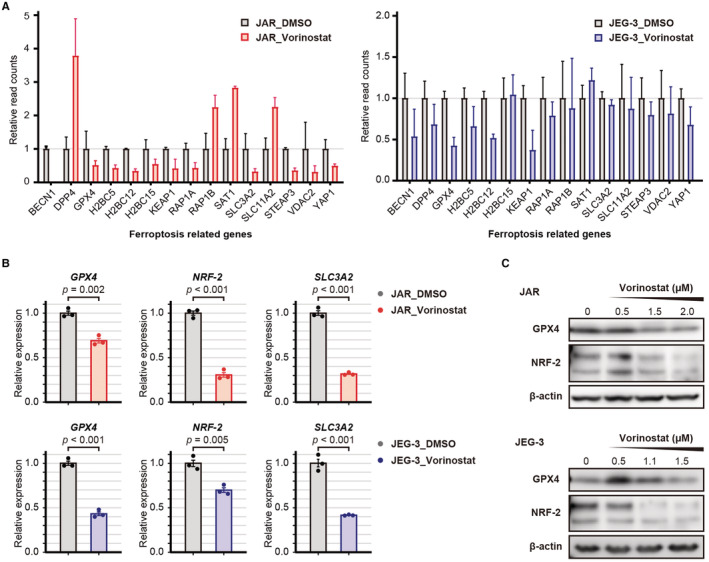
Induction of ferroptosis pathway by vorinostat. (A) The relative read counts of ferroptosis‐associated genes in 1 μM vorinostat‐treated choriocarcinoma cells compared with DMSO treatment. (B) qRT‐PCR showing the relative expression of GPX4, NRF‐2, and SLC3A2 in vorinostat‐treated choriocarcinoma cells compared with DMSO treatment. The relative expression was compared by a Student's *t*‐test, and the values are shown as the mean ± standard error. (C) Western blot analysis of GPX4 and NRF‐2 in choriocarcinoma cell lines treated with vorinostat.

RNA sequencing revealed that vorinostat treatment resulted in the aberrant expression of ferroptosis‐associated genes (Figure 5A). In addition, PCR validated the downregulation of *GPX4*, *NRF2*, and *SLC3A2* in vorinostat‐treated JAR and JEG‐3 cells (Figure 5B). Moreover, western blot analysis revealed that the expression of GPX4 and NRF2 was decreased by vorinostat in a dose‐dependent manner (Figure 5C).

### 3.5 In vivo validation of vorinostat effects

**FIGURE 6:**
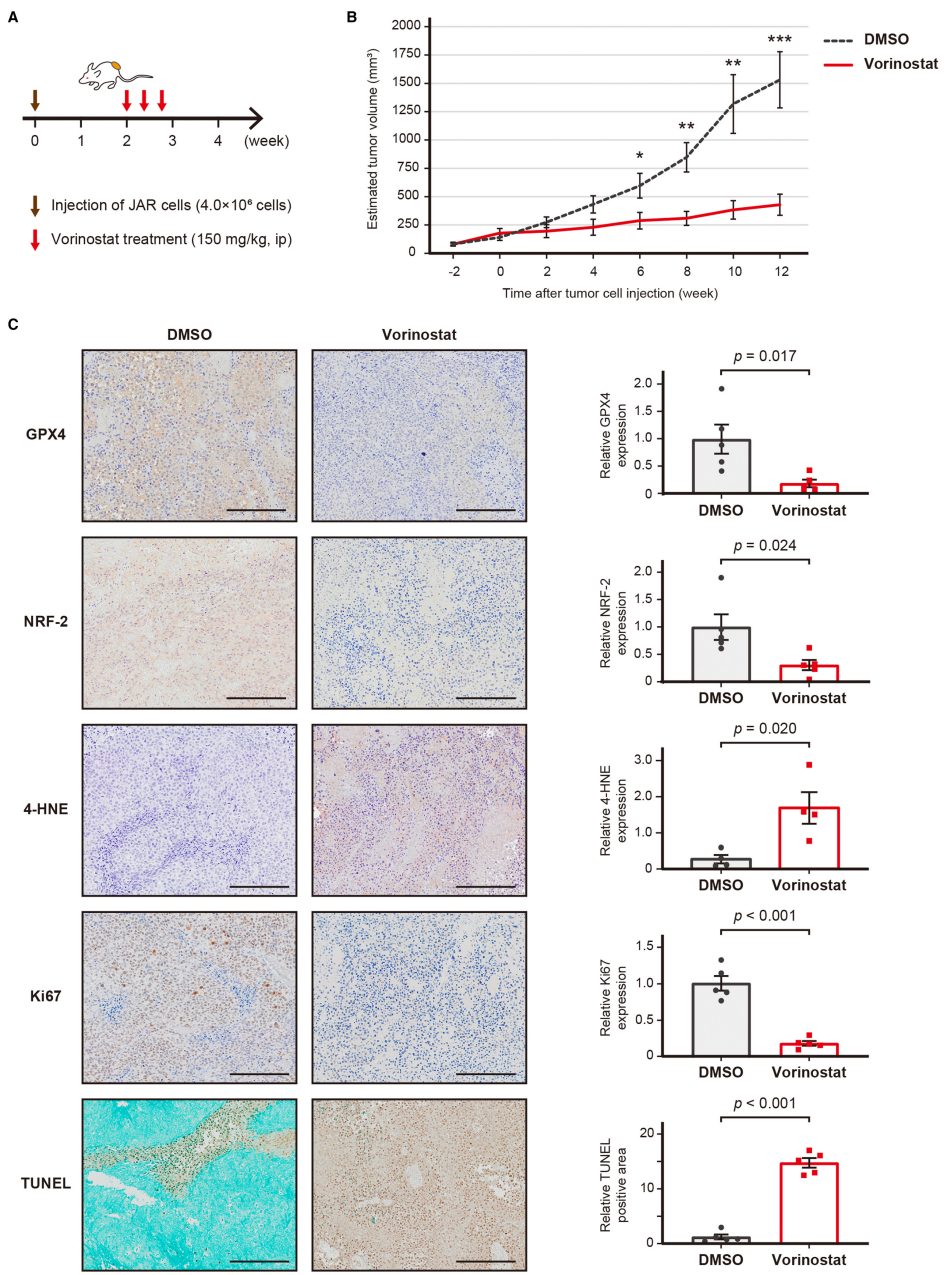
The effect of vorinostat in a JAR cell‐bearing mouse model. (A) The schema of the treatment schedule. Mice were intraperitoneally administered with either vorinostat (150 mg/kg) or vehicle (DMSO) every other day. (B) Estimated tumor volume of JAR cell‐bearing mice treated with either vorinostat or DMSO. Tumor volume and weight were compared using a Student's *t*‐test (*p < 0.05, **p < 0.01, ***p < 0.001). (C) Representative images of immunohistochemistry. The percentage of GPX4‐positive, NRF2‐positive, Ki67‐positive, TUNEL‐positive, and 4HNE‐positive cells were quantified by TissuemorphPD and compared with a Students *t*‐test. The values are the mean ± standard error (*n* = 5). Scale bar, 200 μm.

Finally, we evaluated in vivo effect of vorinostat using a JAR cell‐bearing mouse model (Figure 6A). Tumor volume was significantly inhibited by vorinostat treatment (*p* < 0.01 at 12 weeks) and no adverse events, including weight changes, were observed in either group (Figure 6B). Immunohistochemistry revealed that vorinostat significantly decreased the expression of GPX4 (*p* = 0.017, Figure 6C) and NRF2 (*p* = 0.024, Figure 6C). Additionally, the expression of 4‐hydroxynonena (4‐HNE), a well‐known by‐product of lipid peroxidation and a widely accepted marker of oxidative stress, was increased in the vorinostat group (*p* = 0.020, Figure 6C). Moreover, the expression of Ki67 was significantly decreased (*p* < 0.001) and TUNEL‐positive cells were significantly increased in the vorinostat group (*p* < 0.001, Figure 6C).

## 4 DISCUSSION

In this study, we demonstrated that vorinostat is one of the novel therapeutic agents for choriocarcinoma based on a drug screening strategy. Choriocarcinoma is a rare tumor, and clinical trials are difficult to conduct. Therefore, as a clinically approved antitumor agent, vorinostat has a major advantage over other HDAC inhibitors.^12^ Vorinostat‐induced cell death and decreased the expression of *GPX4*, and *NRF2*, suggesting that ferroptosis was one of the molecular mechanisms underlying the antitumor effects of the drug.

Vorinostat, a potent inhibitor of HDACs class I and II, was the first agent approved by the FDA for the treatment of cutaneous T‐cell lymphoma.^13^ HDACs play important roles in regulating gene expression and modulating various cellular processes. The mechanism of cancer promotion by HDAC involves transcriptional silencing of tumor suppressors through nucleosome deacetylation‐containing tumor suppressor genes.^14^ HDAC inhibitors induce cell cycle arrest and apoptosis in cancer cells. HDAC inhibitors upregulate CDKN1A (p21), a potent cell cycle inhibitor, as well as activate p53 acetylation.^12^, ^15^, ^16^ Moreover, HDAC inhibitors stimulate the transcription of proapoptotic genes, such as BAX, BAK, and APAF1, and inhibit angiogenesis.^17^, ^18^ Thus, several studies have shown that vorinostat exhibits an antitumor effect on lung cancer, prostate cancer, and melanoma.^19^, ^20^, ^21^, ^22^ Moreover, according to subsequent clinical trials, vorinostat demonstrated a high proportion of patients with stable disease, although it failed to exhibit a significant effect.^23^

Ferroptosis is an iron‐dependent form of regulated cell death induced by the accumulation of lipid peroxidation.^24^, ^25^, ^26^ Ferroptosis has been reported to be involved in cancer development in a variety of cancers.^27^ Moreover, ferroptosis was induced in trophoblastic cells in patients with preeclampsia.^28^ Clinically, choriocarcinoma exhibits intratumoral hemorrhage, which suggests the iron‐abundant cancer microenvironment. However, there have been no reports on ferroptosis in choriocarcinoma. *GPX4* and *NRF2* are critical regulators of ferroptosis.^24^, ^29^, ^30^, ^31^ GPX4 utilizes reduced glutathione to convert lipid hydroperoxides to lipid alcohols, resulting in the inhibition of ferroptosis. Therefore, the loss or pharmacological inhibition of GPX4 leads to ferroptosis induction.^30^, ^31^ *NRF2* also has various functions, including glutathione, NADPH, and iron regulation.^32^, ^33^, ^34^ *NRF2* also indirectly regulates lipids, and the amount of NRF2 contributes to ferroptosis susceptibility.^35^ As a result, the activation of NRF2 leads to cancer cell resistance to ferroptosis.^36^, ^37^ Furthermore, recent reports showed that *NRF2* knockdown leads to lower proliferation rates in vitro and in vivo in xenograft experiments of various carcinomas.^38^, ^39^, ^40^ Emerging evidence suggests that ferroptosis is involved in tumorigenesis and chemotherapeutic drug resistance.^27^, ^41^

We demonstrated that an HDAC inhibitor induces ferroptosis in choriocarcinoma cells. This finding is consistent with that of previous reports. Zhang T, et al. demonstrated that vorinostat can promote ferroptosis by inhibiting xCT expression in *EGFR*‐activating mutant lung cancer cells.^42^ xCT is a light chain subunit of the glutamate‐cystine antiporter system, Xc(−), which consists of two subunits, the SLC7A11 light chain subunit and SLC3A2 heavy chain subunit.^43^, ^44^ *SLC7A11* is overexpressed in several cancer types and promotes cystine uptake and glutathione biosynthesis, which resulted in protection from oxidative stress and ferroptosis.^45^, ^46^, ^47^, ^48^ Vorinostat inhibits xCT resulting in the accumulation of oxidative stress and causing ferroptosis in cells.^42^ Our study showed that vorinostat reduced the expression of *SLC3A2*, but not *SLC7A11*. SLC3A2 functions primarily as a chaperone protein and is important for regulating the SLC7A11 transportation to the plasma membrane.^49^ Hence, the loss of SLC3A2 results in a significant reduction in the levels of SLC7A11 protein.^50^ In choriocarcinoma, vorinostat causes a decrease in the level of SLC7A11 protein through a reduction in the expression of SLC3A2, which may lead to deregulated xCT function. As a result, the expression of *GPX4*, a downstream of xCT, was also reduced by vorinostat, which supports this hypothesis.

Our study had several limitations. First, when vorinostat causes cell death in choriocarcinoma cells, it is unclear whether the mechanism is only related to ferroptosis. Wang et al. suggested that the HDAC inhibitor, quisinostat, significantly induced tongue squamous cell carcinoma cell apoptosis and ferroptosis.^51^ In our study, we observed an increase in TUNEL‐positive cells in tumors of mice treated with vorinostat, which may suggest that vorinostat induces both ferroptosis and apoptosis in choriocarcinoma. Moreover, HDAC inhibitors can regulate various gene expressions depending on the cell types. Therefore, the mechanisms of vorinostat will be a subject of further exploration. Secondly, the efficacy of vorinostat by oral administration was not determined in the animal experiments, although vorinostat is clinically an oral drug. However, the safety of vorinostat is clinically assured by the FDA for advanced primary cutaneous T‐cell lymphoma; thus, oral animal experiments are not necessarily required.

In conclusion, our results highlight vorinostat showed a remarkable anticancer effect by regulating the expression of ferroptosis‐related genes. In the future, we will consider conducting a clinical trial of vorinostat in patients with choriocarcinoma.

## AUTHOR CONTRIBUTIONS

Eri Watanabe: conceptualization, data curation, formal analysis, investigation, and writing—original draft. Akira Yokoi: funding acquisition, project administration, supervision, and writing—review and editing. Kosuke Yoshida: data curation, formal analysis, visualization, and writing—review and editing. Mai Sugiyama: investigation. Masami Kitagawa: investigation. Kimihiro Nishino: resources. Eiko Yamamoto: conceptualization and resources. Kaoru Niimi: conceptualization, project administration, resources, and writing—review and editing. Yusuke Yamamoto: resources, Software, visualization. Hiroaki Kajiyama: resources, supervision, and writing—review and editing.

## FUNDING INFORMATION

This study was supported by the following funding: Japan Society for the Promotion of Science Grant‐in‐Aid for Scientific Research: Grant Number 20K22806 and 21H03075; the Fusion Oriented Research for disruptive Science and Technology (FOREST) from the Japan Science and Technology Agency (JST); Practical Research for Innovative Cancer Control, grant number 20ck0106630h0001 from the Japan Agency for Medical Research and Development (AMED); Princess Takamatsu Cancer Research Fund; Aichi Cancer Research Foundation; The Mochida Memorial Foundation for Medical and Pharmaceutical Research.

## CONFLICT OF INTEREST

The authors have no conflict of interest.

## Supporting information


Table S1

Table S2

Table S3

Table S4

Table S5

Table S6

Table S7

## Data Availability

The data generated in this study are publicly available in Gene Expression Omnibus (GEO) at GSE192446.

## References

[1] Lurain JR . Gestational trophoblastic disease I: epidemiology, pathology, clinical presentation and diagnosis of gestational trophoblastic disease, and management of hydatidiform mole. Am J Obstet Gynecol. 2010;203(6):531‐539. 20728069 10.1016/j.ajog.2010.06.073

[2] Berkowitz RS , Goldstein DP . Clinical practice. Molar pregnancy. N Engl J Med. 2009;360(16):1639‐1645. 19369669 10.1056/NEJMcp0900696

[3] Robbins SL , Kumar V , Cotran RS . Robbins and Cotran Pathologic Basis of Disease. Saunders/Elsevier; 2010.

[4] Seckl MJ , Sebire NJ , Berkowitz RS . Gestational trophoblastic disease. Lancet. 2010;376(9742):717‐729. 20673583 10.1016/S0140-6736(10)60280-2

[5] Ngan HY , Kohorn EI , Cole LA , et al. Trophoblastic disease. Int J Gynaecol Obstet. 2012;119(suppl 2):S130‐S136. 22999504 10.1016/S0020-7292(12)60026-5

[6] Powles T , Savage PM , Stebbing J , et al. A comparison of patients with relapsed and chemo‐refractory gestational trophoblastic neoplasia. Br J Cancer. 2007;96(5):732‐737. 17299394 10.1038/sj.bjc.6603608 PMC2360082

[7] Sato S , Yamamoto E , Niimi K , et al. The efficacy and toxicity of 4‐day chemotherapy with methotrexate, etoposide and actinomycin D in patients with choriocarcinoma and high‐risk gestational trophoblastic neoplasia. Int J Clin Oncol. 2020;25(1):203‐209. 31520175 10.1007/s10147-019-01540-9

[8] Zhang J , Yang PL , Gray NS . Targeting cancer with small molecule kinase inhibitors. Nat Rev Cancer. 2009;9(1):28‐39. 19104514 10.1038/nrc2559 PMC12406740

[9] Moffat JG , Vincent F , Lee JA , Eder J , Prunotto M . Opportunities and challenges in phenotypic drug discovery: an industry perspective. Nat Rev Drug Discov. 2017;16(8):531‐543. 28685762 10.1038/nrd.2017.111

[10] Swinney DC . Phenotypic vs. target‐based drug discovery for first‐in‐class medicines. Clin Pharmacol Ther. 2013;93(4):299‐301. 23511784 10.1038/clpt.2012.236

[11] Jones LH , Bunnage ME . Applications of chemogenomic library screening in drug discovery. Nat Rev Drug Discov. 2017;16(4):285‐296. 28104905 10.1038/nrd.2016.244

[12] Takai N , Ueda T , Nishida M , Nasu K , Narahara H . Histone deacetylase inhibitors induce growth inhibition, cell cycle arrest and apoptosis in human choriocarcinoma cells. Int J Mol Med. 2008;21(1):109‐115. 18097623

[13] Kavanaugh SM , White LA , Kolesar JM . Vorinostat: a novel therapy for the treatment of cutaneous T‐cell lymphoma. Am J Health Syst Pharm. 2010;67(10):793‐797. 20479100 10.2146/ajhp090247

[14] Pasini A , Marchetti C , Sissi C , et al. Novel polyamine‐naphthalene diimide conjugates targeting histone deacetylases and DNA for cancer phenotype reprogramming. ACS Med Chem Lett. 2017;8(12):1218‐1223. 29259737 10.1021/acsmedchemlett.7b00289 PMC5733267

[15] Uehara N , Yoshizawa K , Tsubura A . Vorinostat enhances protein stability of p27 and p21 through negative regulation of Skp2 and Cks1 in human breast cancer cells. Oncol Rep. 2012;28(1):105‐110. 22484732 10.3892/or.2012.1758

[16] Kawamata N , Chen J , Koeffler HP . Suberoylanilide hydroxamic acid (SAHA; vorinostat) suppresses translation of cyclin D1 in mantle cell lymphoma cells. Blood. 2007;110(7):2667‐2673. 17606765 10.1182/blood-2005-11-026344 PMC1988938

[17] Marchion D , Münster P . Development of histone deacetylase inhibitors for cancer treatment. Expert Rev Anticancer Ther. 2007;7(4):583‐598. 17428177 10.1586/14737140.7.4.583

[18] Qian DZ , Wang X , Kachhap SK , et al. The histone deacetylase inhibitor NVP‐LAQ824 inhibits angiogenesis and has a greater antitumor effect in combination with the vascular endothelial growth factor receptor tyrosine kinase inhibitor PTK787/ZK222584. Cancer Res. 2004;64(18):6626‐6634. 15374977 10.1158/0008-5472.CAN-04-0540

[19] Kumagai T , Wakimoto N , Yin D , et al. Histone deacetylase inhibitor, suberoylanilide hydroxamic acid (Vorinostat, SAHA) profoundly inhibits the growth of human pancreatic cancer cells. Int J Cancer. 2007;121(3):656‐665. 17417771 10.1002/ijc.22558

[20] Butler LM , Agus DB , Scher HI , et al. Suberoylanilide hydroxamic acid, an inhibitor of histone deacetylase, suppresses the growth of prostate cancer cells in vitro and in vivo. Cancer Res. 2000;60(18):5165‐5170. 11016644

[21] Nervi C , De Marinis E , Codacci‐Pisanelli G . Epigenetic treatment of solid tumours: a review of clinical trials. Clin Epigenetics. 2015;7:127. 26692909 10.1186/s13148-015-0157-2 PMC4676165

[22] Schneider BJ , Kalemkerian GP , Bradley D , et al. Phase I study of vorinostat (suberoylanilide hydroxamic acid, NSC 701852) in combination with docetaxel in patients with advanced and relapsed solid malignancies. Invest New Drugs. 2012;30(1):249‐257. 20686817 10.1007/s10637-010-9503-6

[23] Haas NB , Quirt I , Hotte S , et al. Phase II trial of vorinostat in advanced melanoma. Invest New Drugs. 2014;32(3):526‐534. 24464266 10.1007/s10637-014-0066-9

[24] Stockwell BR , Friedmann Angeli JP , Bayir H , et al. Ferroptosis: a regulated cell death nexus linking metabolism, redox biology, and disease. Cell. 2017;171(2):273‐285. 28985560 10.1016/j.cell.2017.09.021 PMC5685180

[25] Magtanong L , Ko PJ , Dixon SJ . Emerging roles for lipids in non‐apoptotic cell death. Cell Death Differ. 2016;23(7):1099‐1109. 26967968 10.1038/cdd.2016.25 PMC5399169

[26] Dixon SJ , Lemberg KM , Lamprecht MR , et al. Ferroptosis: an iron‐dependent form of nonapoptotic cell death. Cell. 2012;149(5):1060‐1072. 22632970 10.1016/j.cell.2012.03.042 PMC3367386

[27] Toyokuni S , Yanatori I , Kong Y , Zheng H , Motooka Y , Jiang L . Ferroptosis at the crossroads of infection, aging and cancer. Cancer Sci. 2020;111(8):2665‐2671. 32437084 10.1111/cas.14496 PMC7419040

[28] Zhang H , He Y , Wang JX , et al. miR‐30‐5p‐mediated ferroptosis of trophoblasts is implicated in the pathogenesis of preeclampsia. Redox Biol. 2020;29:101402. 31926626 10.1016/j.redox.2019.101402 PMC6928320

[29] Yang WS , Kim KJ , Gaschler MM , Patel M , Shchepinov MS , Stockwell BR . Peroxidation of polyunsaturated fatty acids by lipoxygenases drives ferroptosis. Proc Natl Acad Sci USA. 2016;113(34):E4966‐E4975. 27506793 10.1073/pnas.1603244113 PMC5003261

[30] Yang WS , SriRamaratnam R , Welsch ME , et al. Regulation of ferroptotic cancer cell death by GPX4. Cell. 2014;156(1‐2):317‐331. 24439385 10.1016/j.cell.2013.12.010 PMC4076414

[31] Seibt TM , Proneth B , Conrad M . Role of GPX4 in ferroptosis and its pharmacological implication. Free Radic Biol Med. 2019;133:144‐152. 30219704 10.1016/j.freeradbiomed.2018.09.014

[32] Lee JM , Calkins MJ , Chan K , Kan YW , Johnson JA . Identification of the NF‐E2‐related factor‐2‐dependent genes conferring protection against oxidative stress in primary cortical astrocytes using oligonucleotide microarray analysis. J Biol Chem. 2003;278(14):12029‐12038. 12556532 10.1074/jbc.M211558200

[33] Kerins MJ , Ooi A . The roles of NRF2 in modulating cellular iron homeostasis. Antioxid Redox Signal. 2018;29(17):1756‐1773. 28793787 10.1089/ars.2017.7176 PMC6208163

[34] Wu KC , Cui JY , Klaassen CD . Beneficial role of Nrf2 in regulating NADPH generation and consumption. Toxicol Sci. 2011;123(2):590‐600. 21775727 10.1093/toxsci/kfr183 PMC3179677

[35] Lee C . Collaborative power of Nrf2 and PPARγ activators against metabolic and drug‐induced oxidative injury. Oxid Med Cell Longev. 2017;2017:1378175. 28928902 10.1155/2017/1378175 PMC5591982

[36] Sun X , Ou Z , Chen R , et al. Activation of the p62‐Keap1‐NRF2 pathway protects against ferroptosis in hepatocellular carcinoma cells. Hepatology. 2016;63(1):173‐184. 26403645 10.1002/hep.28251 PMC4688087

[37] Roh JL , Kim EH , Jang H , Shin D . Nrf2 inhibition reverses the resistance of cisplatin‐resistant head and neck cancer cells to artesunate‐induced ferroptosis. Redox Biol. 2017;11:254‐262. 28012440 10.1016/j.redox.2016.12.010 PMC5198738

[38] Ma X , Zhang J , Liu S , Huang Y , Chen B , Wang D . Nrf2 knockdown by shRNA inhibits tumor growth and increases efficacy of chemotherapy in cervical cancer. Cancer Chemother Pharmacol. 2012;69(2):485‐494. 21842204 10.1007/s00280-011-1722-9

[39] Homma S , Ishii Y , Morishima Y , et al. Nrf2 enhances cell proliferation and resistance to anticancer drugs in human lung cancer. Clin Cancer Res. 2009;15(10):3423‐3432. 19417020 10.1158/1078-0432.CCR-08-2822

[40] Lister A , Nedjadi T , Kitteringham NR , et al. Nrf2 is overexpressed in pancreatic cancer: implications for cell proliferation and therapy. Mol Cancer. 2011;10:37. 21489257 10.1186/1476-4598-10-37 PMC3098205

[41] Wu Y , Zhang S , Gong X , et al. The epigenetic regulators and metabolic changes in ferroptosis‐associated cancer progression. Mol Cancer. 2020;19(1):39. 32103754 10.1186/s12943-020-01157-x PMC7045519

[42] Zhang T , Sun B , Zhong C , et al. Targeting histone deacetylase enhances the therapeutic effect of Erastin‐induced ferroptosis in EGFR‐activating mutant lung adenocarcinoma. Transl Lung Cancer Res. 2021;10(4):1857‐1872. 34012798 10.21037/tlcr-21-303 PMC8107764

[43] Sato H , Tamba M , Ishii T , Bannai S . Cloning and expression of a plasma membrane cystine/glutamate exchange transporter composed of two distinct proteins. J Biol Chem. 1999;274(17):11455‐11458. 10206947 10.1074/jbc.274.17.11455

[44] Sato H , Tamba M , Kuriyama‐Matsumura K , Okuno S , Bannai S . Molecular cloning and expression of human xCT, the light chain of amino acid transport system x_c_ . Antioxid Redox Signal. 2000;2(4):665‐671. 11213471 10.1089/ars.2000.2.4-665

[45] Zhang L , Huang Y , Ling J , et al. Overexpression of SLC7A11: a novel oncogene and an indicator of unfavorable prognosis for liver carcinoma. Future Oncol. 2018;14(10):927‐936. 29528250 10.2217/fon-2017-0540

[46] Timmerman LA , Holton T , Yuneva M , et al. Glutamine sensitivity analysis identifies the xCT antiporter as a common triple‐negative breast tumor therapeutic target. Cancer Cell 2013, 24(4):450–465. 24094812 10.1016/j.ccr.2013.08.020 PMC3931310

[47] Ji X , Qian J , Rahman SMJ , et al. xCT (SLC7A11)‐mediated metabolic reprogramming promotes non‐small cell lung cancer progression. Oncogene. 2018;37(36):5007‐5019. 29789716 10.1038/s41388-018-0307-z PMC6127081

[48] Koppula P , Zhang Y , Zhuang L , Gan B . Amino acid transporter SLC7A11/xCT at the crossroads of regulating redox homeostasis and nutrient dependency of cancer. Cancer Commun (Lond). 2018;38(1):12. 29764521 10.1186/s40880-018-0288-x PMC5993148

[49] Nakamura E , Sato M , Yang H , et al. 4F2 (CD98) heavy chain is associated covalently with an amino acid transporter and controls intracellular trafficking and membrane topology of 4F2 heterodimer. J Biol Chem. 1999;274(5):3009‐3016. 9915839 10.1074/jbc.274.5.3009

[50] Shin CS , Mishra P , Watrous JD , et al. The glutamate/cystine xCT antiporter antagonizes glutamine metabolism and reduces nutrient flexibility. Nat Commun. 2017;8:15074. 28429737 10.1038/ncomms15074 PMC5413954

[51] Wang X , Liu K , Gong H , et al. Death by histone deacetylase inhibitor quisinostat in tongue squamous cell carcinoma via apoptosis, pyroptosis, and ferroptosis. Toxicol Appl Pharmacol. 2021;410:115363. 33290780 10.1016/j.taap.2020.115363

